# Value of montelukast as a potential treatment of post-COVID-19 persistent cough: a non-randomized controlled pilot study

**DOI:** 10.1186/s43168-022-00154-6

**Published:** 2022-09-15

**Authors:** Aliae A. R. Mohamed Hussein, Mohamed Eltaher A. A. Ibrahim, Hoda A. Makhlouf, Nahed A. Makhlouf, Howaida K. Abd-Elaal, Karima M. S. Kholief, Islam G. Sayed

**Affiliations:** 1 Pulmonology, Chest Department, Assiut University Hospitals, Assiut, 71515 Egypt; 2 Gastroenterology and Hepatology, Alrajhi Liver Hospital, Assiut University, Assiut, 71515 Egypt; 3 Internal Medicine and Surgery (adults), Faculty of Nursing, Assiut University, Assiut, Egypt; 4 Public Health and Community Medicine Department, Assiut Faculty of Medicine, Assiut, Egypt; 5 Pulmonology, Aswan Faculty of Medicine, Aswan, Egypt

**Keywords:** Montelukast, COVID-19, SARS-CoV-2, Post COVID-19, Cough, Cough severity

## Abstract

**Background:**

This pilot study included 68 cases with post-COVID-19 persistent cough (> 8 weeks), randomly allocated into two groups; intervention group (32 patients) received standard cough therapy, and montelukast 10 mg/day for 14 days and control group (36 patients) received only cough sedatives.

**Results:**

We found a significant improvement in the number of cough paroxysms/day, cough severity visual analog scale, cough severity index and cough quality of life, shorter duration improvement, and minimal side effects in the interventional group.

**Conclusions:**

We suggest that montelukast may be effective to reduce the duration and severity of the persistent post-COVID-19 cough and further improve quality of life.

## Background

As of September 2020, the COVID-19 contagion has affected millions of people in several countries and left hundreds of thousands deceased. After convalescence from the acute attack, it was found that up to 32% of cases had 1 or 2 symptoms, 55% had 3 or more post-COVID-19 complaints [1], and persistent post-COVID-19 cough was documented in 29.3% of cases in a previous study [2].

A recent study acknowledged montelukast, among the top-scoring clinically oriented drugs probably to hinder new corona virus main protease [3]. Besides its known effect that is reported to ameliorate cough [4] and halt exercise-induced bronchoconstriction in asthmatic patients [5], many trials evaluated montelukast in the management of post-contagious cough and disclosed variable effects [6, 7].

**Fig. 1:**
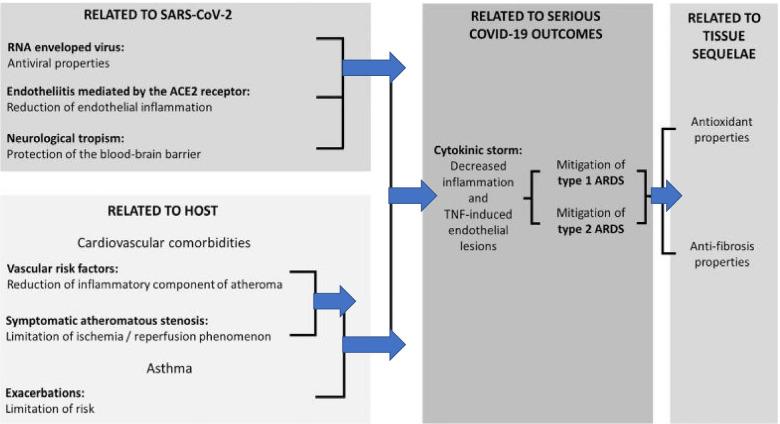
Experimentally supported properties of cyst LT1 receptor antagonists potentially beneficial in COVID-19 (adapted from Barré et al. 2020)

Despite that the exact mechanism is not yet identified, Barré and colleagues anticipated several properties of CystLT1 receptor antagonists that were potentially beneficial in COVID-19 cases as demonstrated in Fig. 1 [8].

*The aim of this pilot study* was to assess the effect of 2-week treatment with montelukast on both the cough severity and cough-related quality of life among patients with persistent post-COVID-19 cough.

## Patients and methods

This interventional open-label non-randomized controlled pilot trial was conducted in post-COVID-19 outpatient clinics in a tertiary hospital. Recovered cases with confirmed COVID-19 were recruited. (Since many people were not tested, and false-negative tests are common, it was suggested that a positive test for COVID-19 is not a prerequisite for diagnosis.)

In the absence of agreed definitions, for the purposes of this study, Assaf et al. defined post-acute COVID-19 as extending symptoms beyond 21 days from the onset of first complaints and chronic COVID-19 as extending beyond 3 months [9]. The British Thoracic Society defined chronic cough as one that continues beyond 8 weeks [10]. Up to that time, and unless there are super-infection signs or other *obstacles* such as throbbing pleural inflammation, it is proposed that cough is best managed with simple breathing control exercises and drugs when recommended (such as proton-pump inhibitors if reflux is supposed) [11].

### Intervention

The pilot study included 426 patients with confirmed COVID-19. Of 126 (26.5%) cases with persistent cough (> 8 weeks), 58 cases were excluded (as 21 cases were previously diagnosed with asthma, 19 COPD, 18 GERD), and the remaining were randomly allocated into 2 groups (cross over 1:1): intervention group (32 patients) received standard cough therapy+ montelukast 10 mg/day for 14 days, and control group (36 patients) received only cough therapy. The CT chest findings in the studied groups were unremarkable.

### Baseline evaluation

During the period of follow-up, complete history taking, and physical examination, a CT chest was performed to exclude any allied persistent ground-glass opacities or fibrosis, asthma, COPD, and GERD, and prior cough *medicaments* in the last 2 weeks (including antibiotic, antihistamine, dextromethorphan, and codeine-based agents) were recorded.

At day 0 and day 14 of intervention, the following were assessed for each patient:

1. Cough parameters comprising cough duration, number of paroxysms/days, cough severity index, cough visual analog scale (VAS), and cough quality-of-life questionnaire [12, 13]
2. Drug-related side effects were documented at the end of 14 days.

### Exclusion criteria

It is any contraindication to montelukast therapy, respiratory, cardiac illness, pregnancy, breastfeeding, and use of angiotensin-converting enzyme inhibitors.

This study was approved by the Faculty Research Ethics Committee; every case gave written informed consent.

## Results

This pilot study included 426 patients with confirmed COVID-19 (PCR and/or clinical radiologic laboratory), male/female 154/272 (36/64%), their mean age was 43 ± 12 (19–73) years old, 367 (86%) were nonsmokers, only 103 (24.1%) received influenza vaccine in the preceding year, 162 (38%) were PCR positive, 115 (26.9%) had comorbidities, and 101 (23.7%) required hospital admission. During acute COVID-19, they were treated by antibiotics (86.6%), hydroxychloroquine (26.5%), steroids (44.1%), anticoagulants (48.5%), and 100% vitamins and zinc. The mean duration of recovery was 65 ± 18 (14–120) days.

From 126 (26.5%) cases with persistent cough (> 8 weeks), 21 cases were excluded (as they were previously diagnosed as asthma, 19 COPD, 18 GERD); the remaining were randomly allocated into two groups (cross over 1:1): intervention group 32 patients received standard cough therapy+ montelukast 10 mg/day for 14 days, and control group 36 patients received cough therapy. Their CT chest findings were unremarkable.

Table 1 showed that after 14-day treatment with montelukast 10 mg daily, there was a considerable improvement in number of paroxysms/days in interventional group (*P* < 0.01), cough severity VAS (*P* < 0.001), cough severity index (*P* < 0.01), and cough quality of life (*P* < 0.01). Mean numbers of days needed for improvement were 5 ± 1.4 in interventional vs. 10 ± 1.5 days in noninterventional group (*P* < 0.01), and the side effects were recorded in 18.7% of cases.

**Table 1:** Cough severity in patients with post-COVID-19 cough measured by number of paroxysms, visual analogue scale (VAS), cough severity index, and cough quality-of-life questionnaire after 14-day treatment with montelukast 10 mg (*n* = 68 patients)

	Cough severity in day 1 in study groups (*n* = 68)	Cough severity after 14-day montelukast therapy* (*n* = 32)	Cough severity in controls after 14 days standard therapy# (*n* = 36)	*p*-value
Number of paroxysms/days	12 ± 3.1	3 ± 1.2*	10 ± 4.1#	*#< 0.01
Cough severity VAS	76 ± 19	12 ± 6*	66 ± 12#	< 0.001
Cough severity index	21 ± 2	4 ± 1.1*	20 ± 5#	< 0.01
Cough quality of life questionnaire	87 ± 4.9	18 ± 2.5*	98 ± 2#	< 0.01
Days needed for improvement	-	5 ± 1.4	10 ± 1.5#	#< 0.05
Side effects^a^	-	6 (18.7)	-	N/A

Data were expressed as number (%), mean ± SD. *VAS* visual analogue scale (100 mm); cough VAS: 0 = no cough, 100 = worst cough ever. *Variance before and after montelukast, #variance between intervention group and controls.

^a^Upper respiratory contagion (infection in the nose or throat), fever, headache, sore throat, cough, stomach pain, diarrhea, earache, or ear infection

## Discussion

Post-contagious cough is common in primary care but has no confirmed effective medications. Cysteinyl leukotrienes are enrolled in the immunopathogenesis of post-contagious cough, and montelukast, a cysteinyl leukotriene receptor antagonist, may be efficacious in the management of post-contagious cough [12]. The main finding of this report is that montelukast had a considerable effect in reducing cough among patients with persistent post-COVID-19 cough as assessed by number of cough paroxysms/day (*P* < 0.01), cough severity by VAS (*P* < 0.001), cough severity index (*P* < 0.01), and cough quality of life (*P* < 0.01). The mean number of days needed for improvement was 5 ± 1.4 vs. 10 ± 1.5 days in interventional vs. non-interventional group (*P* < 0.01), and the side effects were recorded in 18.7% of cases. To our knowledge, we report the first case-control randomized trial of montelukast for the management of post-COVID-19 persistent cough.

This report showed that the frequency of post-COVID-19 persistent cough was 26.5%. Similar data of persistent post-COVID-19 cough was recorded in 29.3% of the cases in a recent study [2]. The British Thoracic Society defines chronic cough as one that continues beyond 2 months [10]. As for management, it was specified that unless there are signs of super contagion or other complications for instance painful pleurisy, cough seems to be best managed with simple breathing control exercises and medications if indicated (for example, proton-pump inhibitors, if reflux is assumed) [11].

The recorded considerable improvements in the severity of cough in the present study were in accordance with many reports finding improvements in cough-specific quality of life in the treatment groups [13, 14]. However, others found that montelukast was not an effective therapy for post-contagious cough and explained the improvement by the self-limited nature of post-contagious cough [12]

A recent retrospective study consistently disclosed that elder asthmatic patients taking montelukast had less incidents of confirmed COVID-19 than those not [15]. It was proposed that montelukast has a role on events evolving with ACE receptors and also has an anti-inflammatory reaction with bradykinin and leukotriene antagonism. As COVID-19 has entry into the cell through ACE receptors and caused mortality due to disproportionate inflammatory procedures, it was thought that montelukast may have an effect on the progression of the illness on COVID-19 contagion [16]. They proposed three distinct mechanisms to support the beneficial role of montelukast in the treatment of viral contagions: first, a direct antiviral activity, second, as an antagonist of the cytokine storm, and third by inhibition of the vertical spread and neuroprotective sequelae on the fetal brain [17]. Montelukast convinced a dose-dependent decline in the levels of RNAs expressed, signifying an inhibition of viral replication among hepatitis C patients [18]. It also attenuated the preliminary reactions and sequalae of reinfection to respiratory syncytial virus (RSV) contagion [19, 20]. It was claimed that montelukast may constitute several synergetic and enhancing therapeutic probabilities in COVID-19 (Fig. 1) [8].

Finally, 18.7% of patients in the current study recorded side effects. The drug is generally safe, frequently used, and does not require any prior cardiac or laboratory investigations; it can be prescribed for pregnant women, children, and elders [6]. It may be more effective for patients with comorbidities such as asthma, diabetes, sleep apnea, smoking, obesity, or symptomatic atherosclerotic lesions [7].

## Conclusions

Montelukast may be effective to use to reduce duration, severity of persistent post-COVID-19 cough, and improve quality of life. Further studies on its effect during the course of the illness and recovery are needed. The exact mechanism of montelukast in acute and chronic stages of COVID-19 is yet to be investigated. We support the conduct of several clinical trials testing its effect in COVID-19 cases from a variety of populations, with diverse concentrations while keeping in mind its adverse properties.

## Data Availability

The datasets used and/or analyzed during the current study are available from the corresponding author on reasonable request.

## Abbreviations

VAS: Cough visual analog scale
RSV: Respiratory syncytial virus
ACE: Angiotensin-converting enzyme

## References

[1] Carfì A, Bernabei R, Landi F (2020). For the gemelli against COVID-19 post-acute care study group. Persistent symptoms in patients after acute COVID-19. JAMA..

[2] Galal I, Hussein AA, Amin MT, Saad MM, Zayan HEE, Abdelsayed M Z, ... & Hashem M. K (2021) Determinants of persistent post-COVID-19 symptoms: value of a novel COVID-19 symptom score. Egypt J Bronchol 15(1):1–8

[3] Huynh T, Wang H, Luan B (2020). In silico exploration of the molecular mechanism of clinically oriented drugs for possibly inhibiting SARS-CoV-2’s main protease. J Phys Chem Lett.

[4] Spector SL, Tan RA (2004). Effectiveness of montelukast in the treatment of cough variant asthma. Ann Allergy Asthma Immunol.

[5] Peroni DG, Pescollderungg L, Sandri M, Chinellato I, Boner AL, Piacentini GL (2011). Time-effect of montelukast on protection against exercise-induced bronchoconstriction. Respir Med.

[6] Zhu LQ, Kuang JL, Deng ZC (2011). Clinical analysis of montelukast in the treatment of post-infectious cough. China Pharmacy.

[7] Bisgaard H, Flores-Nunez A, Goh A, Azimi P, Halkas A, Malice MP, Marchal JL, Dass SB, Reiss TF, Knorr BA (2008). Study of montelukast for the treatment of respiratory symptoms of post–respiratory syncytial virus bronchiolitis in children. Am J Respir Crit Care Med.

[8] Barré J, Sabatier JM, Annweiler C (2020). Montelukast drug may improve COVID-19 prognosis: a review of evidence. Front Pharmacol.

[9] Assaf G, Davis H, McCorkell L et al (2020) An analysis of the prolonged COVID-19 symptoms survey by patient-led research team. Patient Led Res https://patientresearchcovid19.com/

[10] British Thoracic Society (2020). British Thoracic Society guidance on respiratory follow up of patients with a clinico-radiological diagnosis of COVID-19 pneumonia.

[11] Homerton University Hospital (2020). Post COVID-19 patient information pack.

[12] Shembel AC, Rosen CA, Zullo TG, Gartner-Schmidt JL (2013) Development and validation of the cough severity index: a severity index for chronic cough related to the upper airway. Laryngoscope. 10.1002/lary.23916 10.1002/lary.23916 23737389

[13] French C, Irwin RS, Fletcher KE (2002). Evaluation of a cough-specific quality-of-life questionnaire. Chest..

[14] Wang K, Birring SS, Taylor K, Fry NK, Hay AD, Moore M, Jin J, Perera R, Farmer A, Little P, Harrison TG (2014). Montelukast for postinfectious cough in adults: a double-blind randomised placebo-controlled trial. The lancet. Respir Med.

[15] Ponsioen BP, Hop WC, Vermue NA, Dekhuijzen PN, Bohnen AM (2005). Efficacy of fluticasone on cough: a randomised controlled trial. Eur Respir J.

[16] Woodcock A, McLeod RL, Sadeh J, Smith JA (2010). The efficacy of a NOP1 agonist (SCH486757) in subacute cough. Lung..

[17] Bozek A, Winterstein J (2020) Montelukast’s ability to fight COVID-19 infection. J Asthma 1–2. 10.1080/02770903.2020.1786112 10.1080/02770903.2020.1786112 32586154

[18] Fidan C, Aydoğdu A (2020). As a potential treatment of COVID-19: montelukast. Med Hypotheses.

[19] Chen Y, Li Y, Wang X, Zou P (2020). Montelukast, an anti-asthmatic drug, inhibits Zika virus infection by disrupting viral integrity. Front Microbiol.

[20] Ruiz I, Nevers Q, Hernández E, Ahnou N, Brillet R, Softic L, ... & Ahmed-Belkacem A (2020) MK-571, a cysteinyl leukotriene receptor 1 antagonist, inhibits hepatitis C virus replication. Antimicrob Agents Chemother 64(6):e02078–19 10.1128/AAC.02078-19 PMC7269486 32179525

